# Seroprevalencia y factores de riesgo asociados a toxoplasmosis gestacional en el Nororiente Colombiano*

**DOI:** 10.15649/cuidarte.2287

**Published:** 2023-05-27

**Authors:** Denny Miley Cárdenas-Sierra, Camila Domínguez-Julio, María Ximena Blanco-Oliveros, Javier Andrés Soto, Elizabeth Tórres-Morale

**Affiliations:** 1 . Investigadora MSc. Universidad de Santander, Facultad de Ciencias Médicas y de la Salud, Instituto de Investigación Masira; Cúcuta, Colombia. Email: de.cardenas@mail.udes.edu.co Universidad de Santander Universidad de Santander Facultad de Ciencias Médicas y de la Salud Instituto de Investigación Masira Cúcuta Colombia de.cardenas@mail.udes.edu.co; 2 . Bacterióloga y Laboratorista Clínico. Universidad de Santander, Facultad de Ciencias Médicas y de la Salud, Instituto de Investigación Masira, Cúcuta, Colombia. Email: cami0808@hotmail.com Universidad de Santander Universidad de Santander Facultad de Ciencias Médicas y de la Salud Instituto de Investigación Masira Cúcuta Colombia cami0808@hotmail.com; 3 . Bacterióloga y Laboratorista Clínico. Universidad de Santander, Facultad de Ciencias Médicas y de la Salud, Instituto de Investigación Masira, Cúcuta, Colombia. Email: ximeblanco63@gmail.com Universidad de Santander Universidad de Santander Facultad de Ciencias Médicas y de la Salud Instituto de Investigación Masira Cúcuta Colombia ximeblanco63@gmail.com; 4 . Investigador PhD. Universidad de Santander, Facultad de Ciencias Médicas y de la Salud, Instituto de Investigación Masira, Cúcuta, Colombia. Email: jav.soto@mail.udes.edu.co Universidad de Santander Universidad de Santander Facultad de Ciencias Médicas y de la Salud Instituto de Investigación Masira Cúcuta Colombia jav.soto@mail.udes.edu.co; 5 . Investigadora MSc. Universidad de Quindío, Facultad de Ciencias de la Salud, Grupo de Investigación Gepamol, Armenia, Quindío. Colombia. Email: etorres@uniquindio.edu.co Universidad del Quindío Universidad de Quindío Facultad de Ciencias de la Salud Armenia Quindío Colombia etorres@uniquindio.edu.co

**Keywords:** Factores de riesgo, Inmunidad, Mujeres embarazadas, Seroprevalencia, Toxoplasmosis, Risk Factors, Immunity, Pregnant Women, Seroprevalence, Toxoplasmosis, Fatores de Risco, Imunidade, Gestantes, Soroprevalência, Toxoplasmose

## Abstract

**Introducción:**

La toxoplasmosis es una zoonosis prevalente en un tercio de la población mundial, que afecta negativamente la salud materno-fetal causando daños de grado variable al feto.

**Objetivo:**

Se propuso evaluar el estado serológico IgG e IgM anti-*Toxoplasma gondii* y factores de riesgo relacionados, en mujeres gestantes de primer trimestre en Cúcuta, Colombia, en el año 2018.

**Material y Métodos:**

Estudio transversal y correlacional en 111 mujeres participando voluntariamente, a quienes se testeó para IgM e IgG específicas por inmunoensayo LIA.

**Resultados:**

Se halló 19,8% y 35,1% de seropositividad total para IgM e IgG, respectivamente, 11,7% lo fue únicamente para IgM y 53,2% corresponde a la frecuencia de seronegatividad global para *T.gondii*; Se identificaron factores de riesgo (IC=95%) como consumo de carne mal cocida (54,1% de los casos, OR=1,8, p=0,120), de agua del grifo (48,6%, OR=1,4, p=0,421), de leche cruda de cabra o de vaca (39,6%, OR=0,78, p=0,553), además de convivencia con gatos (23,4%), éste último asociado significativamente a seropositividad al parásito (OR=2,8, p=0,025).

**Discusión y conclusiones:**

Nuestros hallazgos revelan un posible riesgo de primo-infección en más de la mitad de la población gestante dada su seronegatividad frente al parásito, pero también una frecuencia considerable de casos con sospecha de infección muy reciente, lo que además de asociarse a un factor de riesgo previamente reconocido, deja entrever otros aspectos de riesgo en torno a la alimentación que deben impactarse mediante estrategias de prevención durante el control prenatal, sugiriendo la necesidad de fortalecer la vigilancia en torno al evento.

## Abstract (en)

**Introduction:**

Toxoplasmosis is a zoonosis prevalent in one-third of the world’s population, adversely affecting maternal-fetal health by causing varying degrees of damage to the fetus. Objective: To assess the seroprevalence of *Toxoplasma gondii* IgG and IgM antibodies and associated risk factors in first- trimester pregnant women in Cúcuta, Colombia, in 2018.

**Materials and Methods:**

Cross-sectional and correlational study in 111 women who voluntarily participated. Specific IgM and IgG antibodies were detected by luminescence immunoassay (LIA).

**Results:**

Total seropositivity for IgM and IgG was 19.8% and 35.1%, respectively, 11.7% was for IgM only, and 53.2% corresponded to the overall seronegativity rate for *T. gondii*. Risk factors (CI = 95%) such as undercooked meat consumption (54.1% of cases, OR = 1.8, p = 0.120), tap water consumption (48.6%, OR = 1.4, p = 0.421), and goat or cow raw milk consumption (39.6%, OR = 0.78, p = 0.553) were identified; in addition, living with cats (23.4%) was identified as a risk factor significantly associated with parasite seropositivity (OR = 2.8, p = 0.025).

**Discussion and conclusions:**

Our findings showed a possible risk of primary infection in more than half of the pregnant population, given the seronegativity against the parasite. A considerable frequency of suspected cases of very recent infection was also found. In additionto being associated with a previously recognized risk factor, this fact suggests the presence of other dietary risks that should be addressed through prevention strategies during prenatal care and the need to strengthen event surveillance.

## Resumo

**Introdução:**

A toxoplasmose é uma zoonose prevalente em um terço da população mundial, que afeta negativamente a saúde materno-fetal, causando diversos graus de danos ao feto.

**Objetivo:**

Propôs-se avaliar o estado sorológico IgG e IgM anti - *Toxoplasma gondii* e fatores de risco relacionados, em gestantes no primeiro trimestre em Cúcuta, Colômbia, no ano de 2018.

**Material e Métodos:**

Estudo transversal e correlacional em Participaram 111 mulheres voluntariamente, que foram testadas para IgM e IgG específicos pelo imunoensaio LIA. Resultados: 19,8% e 35,1% do total de soropositividade foram encontrados para IgM e IgG, respectivamente, 11,7% foi apenas para IgM e 53,2% corresponde à frequência de soronegatividade geral para *T.gondii* ; Foram identificados fatores de risco (IC=95%) , como consumo de carne mal cozida (54,1% dos casos, OR= 1,8, p=0,120 ), água da torneira (48,6%, OR=1,4, p=0,421), cru leite de cabra ou vaca (39,6%, OR=0,78, p=0,553 ), além de conviver com gatos (23,4%), sendo este último significativamente associado à soropositividade ao parasita ( OR=2,8 , p =0,025 ).

**Discussão e conclusões:**

Nossos achados revelam um possível risco de infecção primária em mais da metade da população gestante dada a sua soronegatividade contra o parasita, mas também uma frequência considerável de casos com suspeita de infecção muito recente, que, além de estar associada a um fator de risco previamente reconhecido , sugere outros aspectos de risco em torno da alimentação que devem ser impactados por meio de estratégias de prevenção durante o pré-natal, sugerindo a necessidade de fortalecer a vigilância em torno do evento.

## Introducción

*Toxoplasma gondii* es un parásito protozoario intracelular responsable de la infección de un tercio de la población mundial^1^, que puede comprometer significativamente la salud de ciertos subgrupos de la población como las mujeres embarazadas e individuos inmunosuprimidos. Los hospederos definitivos de este microorganismo son los felinos, actuando el hombre y otros mamíferos como hospederos intermediarios.

Los felinos alojan al *Toxoplasma gondii* en la mucosa intestinal y lo expulsan a través de sus heces en una proporción de 10 millones de ooquistes diarios, adquiriéndose la infección de varias maneras, principalmente por vía oral a través del consumo de ooquistes excretados por el gato u otros felinos, o bien de quistes tisulares de huéspedes intermediarios al ingerir carne mal cocida^2^^-^^4^. El consumo de leche de vaca o de cabra cruda (no pasteurizada), carne y verduras crudas o agua contaminada con ooquistes, no tratada^3^^,^^5^, el bajo nivel socioeconómico^6^^,^^7^, vivir en zonas rurales o las labores que implican contacto con el suelo, se han considerado factores de riesgo^8^^,^^9^.

Esta amplia gama de formas de infección pone de manifiesto la búsqueda del parásito en los productos de consumo diario, incluidos los derivados de la carne, buscando identificar el microorganismo en diferentes tipos de fuentes cárnicas y no sólo en el ganado, que es considerado el principal. Un meta- análisis ha descrito la presencia de *Toxoplasma gondii* en otros grupos de animales además del ganado vacuno como cerdos, ovejas, cabras y caballos. Una observación interesante fue que la prevalencia del parásito en el ganado bovino era menor que la identificada en cerdos y ovejas, y que estas tasas tienen una relación directa con el origen geográfico de los animales evaluados^10^.

Se ha referido una seroprevalencia global de IgG anti-*Toxoplasma gondii* del 30-50% de la población^11^^-^^13^ humana, que actualmente abarca incluso un rango más amplio que va desde el 1% hasta el 100% de la población, en base a diversos factores determinantes de índole ambiental y socioeconómico, como: hábitos alimenticios, prácticas higiénicas, susceptibilidad del huésped, localización geográfica y humedad del suelo, siendo por tanto más alta en zonas de clima cálido y húmedas, con una distribución diferencial tanto por edad (mayor seroprevalencia en población de mayor edad)^14^, como por genotipo del parásito, encontrándose los tipos I y III en América del Sur y el tipo II en Europa^15^^-^^18^.

Teniendo en cuenta que la transmisión materno-fetal ocurre preferentemente durante la fase aguda de infección primaria en la madre^19^, es relevante la tamización de toxoplamosis gestacional que busque mitigar la enfermedad congénita relacionada, ya que Colombia, entre otros países, posee una política de control prenatal que incluye un tratamiento oportuno a partir de la determinación de seroconversión^20^^-^^23^, reconociéndose además la importancia de la educación como estrategia de prevención^24^^,^^25^.

Teniendo en cuenta la problemática de salud pública que representa la toxoplasmosis gestacional^26^, es necesario identificar eficazmente los factores de riesgo contextualizados localmente para reducir las tasas de incidencia en los subgrupos más susceptibles, tal como lo son las mujeres gestantes.

Por lo tanto, se propuso a través de la presente investigación evaluar el perfil serológico de esta población, en base a IgG e IgMespecíficas, para establecer conexión conlos factores de riesgo evaluados en este estudio, entendiendo, para efectos de alcance, que la IgM, pese a considerarse en general como un marcador de contacto reciente hospedero-patógeno, en el caso de la toxoplasmosis puede permanecer elevada un lapso prolongado de tiempo, no permitiendo una predicción exacta acerca del tiempo de infección de la gestante, por lo que indica un contacto reciente (no necesariamente infección activa), resaltándose la importancia de su determinación conjunta con IgG^27^^,^^28^.

## Materiales y Métodos

Estudio transversal y correlacional que buscó establecer la asociación entre la respuesta humoral a *T.gondii*, y factores considerados de riesgo de infección, en población de zona fronteriza nororiental colombiana.

La investigación se llevó a cabo por medio de fases o etapas que facilitaron el proceso a seguir:

*Fase descriptiva:* Esta fase consistió en la elaboración y validación del documento de recolección de datos (encuesta) y del consentimiento informado.

*Fase interactiva:* coordinación, conjuntamente con la Institución Prestadora de Servicios de Salud, para el establecimiento de lugar, fecha y hora para entrevista con mujeres de primer trimestre de gestación; enrolamiento de participantes e implementación, tanto del instrumento tipo encuesta como del consentimiento informado.

*Fase analítica:* Obtención de muestra sanguínea (previa firma de consentimiento informado), separación de suero, transporte al Laboratorio de Investigación en la Universidad de Santander y almacenamiento a -80°C hasta procesamiento por serología (como se especificará más adelante). Los resultados fueron registrados en una base de datos con acceso restringido al personal del equipo investigador y con protección de datos personales de las participantes mediante la asignación de un código interno, el cual permitió también garantizar la objetividad de los análisis.

*Fase de informe:* en esta fase se acudió a la Institución de Salud para entrega periódica de resultados del bioanálisis a profesional coordinadora del Programa de Control Prenatal (retroalimentación de información).

### Población

Se contó con una muestra consecutiva de 111 participantes^29^, que incluyó a todas las gestantes de primer trimestre de embarazo que llegaron a su tamizaje inicial de laboratorio, durante el periodo de julio a octubre de 2018, como parte del Programa de Control Prenatal en la Institución de Salud vinculada al estudio, mediante muestreo intencional (por conveniencia), basado en la implementación de criterios de inclusión y exclusión; previo consentimiento informado. Los criterios de inclusión fueron: No tener alguna enfermedad de base inmunológica, ser mayor de edad, ser parte del Programa de Control Prenatal, encontrarse dentro del primer trimestre de gestación y participar de manera completamente voluntaria. Los criterios de exclusión fueron: ser menor de edad, no encontrarse dentro del primer trimestre de gestación, no estar adscrita al Programa de Control Prenatal de la Institución de Salud vinculada al estudio y desistir de participar al principio o durante el estudio.

### Consideraciones éticas:

La presente investigación estuvo en concordancia con los principios éticos para investigaciones médicas en seres humanos establecidos por la Declaración de Helsinki (2013), en lo referente a aspectos como riesgos, confidencialidad, protocolo de investigación y comité de ética de investigación. Implicó riesgo mínimo para las participantes, representado en una punción venosa realizada por personal de salud calificado y en Laboratorio Clínico, con observación de las participantes durante y después del procedimiento e indagación sobre su bienestar, en tanto que se preservó en todo momento la confidencialidad de la información personal de las participantes. Tanto el consentimiento informado como el protocolo de investigación fueron avalados por el Comité de Ética de la E.S.E Imsalud, según Acta 303-791del 24 de mayo de 2018, entidad pertinente e independiente de los investigadores, transparente en su funcionamiento. Se procedió en acuerdo al principio de beneficencia mediante reporte inmediato de resultados positivos ante las autoridades respectivas (Coordinación de Control Prenatal), bajo estricta confidencialidad y propendiendo por el manejo oportuno de las participantes, según los procedimientos administrativos establecidos en dicha Institución y entidades aliadas para el abordaje integral de las mismas.

### Recolección de datos

Se empleó un formulario tipo encuesta validado por dos expertos en el tema (Ginecobstetricia y Enfermería), conformado por tres secciones: datos personales, sociodemográficos (estrato socioeconómico, nivel educativo) y factores relacionados a riesgo de infección por *T.gondii* (variables): convivencia con gatos (y cantidad), consumo de carne mal cocida, consumo de leche cruda (no pasteurizada/hervida) de vaca o cabra, ocupación agrícola, acceso a acueducto/alcantarillado y tipo de agua de consumo (directa del grifo, hervida, envasada o de aljibe).

### Análisis serológico

Se empleó el kit comercial Recomline TORCH Screening (Mikrogen Diagnostik, Germany), basado en el empleo de un lisado de células completas y de antígenos recombinantes previamente fijados en una membrana de nitrocelulosa, para la determinación de anticuerpos IgG e IgM contra *Toxoplasma gondii*, en suero o plasma humano. El análisis de detección de IgM específica incluyó una única banda correspondiente al antígeno de fase temprana ROP1c de *T. gondii*, el cual permite establecer posibilidad de infección alrededor de las últimas ocho semanas. El análisis de detección de IgG específica incluyó una banda de antígeno de lisado de células completas altamente purificado y una banda adicional de antígeno p30 de *T. gondii*.

Se procedió según las indicaciones de la casa comercial; cada muestra de suero diluido se puso en contacto con la totalidad de los antígenos previamente fijados en el soporte de nitrocelulosa por espacio de 1 hora a temperatura ambiente. Luego de tres lavados se adicionó solución de conjugado (anti-IgG ó IgM humanas-HRP) y se incubó de la misma manera por 45 minutos. Posterior a tres lavados se adicionó solución de substrato cromogénico durante 8 minutos. La aparición de una banda oscura en cada zona de reacción indicó positividad, parámetro establecido mediante el uso de escáner con software *Recom*Scan (Mikrogen Diagnostic, Germany), el cual mide la intensidad de las bandas en pixeles, normaliza y considera un resultado positivo a todo aquel cuya intensidad es mayor a la obtenida para la banda de punto de corte o *cut-off*.

### Análisis estadístico

A partir del instrumento de recolección de datos se estableció una base de datos con el fin de tabular toda la información recolectada. Se realizó análisis estadístico basado en la elaboración de distribuciones de frecuencia simple, tablas de contingencia para el cruce de variables y el cálculo de medidas descriptivas, como promedio, media, desviación estándar y rango; para la asociación entre seropositividad a inmunoglobulina G y exposición a factores de riesgo se calculó valor OR con intervalo de confianza (IC) del 95% mediante regresión logística con análisis univariado de variables relevantes, empleando software SPSS v24.

## Resultados

Descripción de la población. Se contó con la participación de 111 mujeres gestantes de primer trimestre de embarazo con una media de 22 años (rango: 18-41), las cuales acudieron al Programa de Control Prenatal en una Institución de Salud de la ciudad de Cúcuta en el nororiente colombiano. Las características de la población se describen en la Tabla 1. (Datos perdidos = 9 por no respuesta a ítem de encuesta, n = 102).

**Tabla 1:** Caracterización de la población gestante de la Institución de Salud vinculada al estudio.

Variable		Frecuencia Absoluta (#)	Frecuencia Relativa (%)
Edad (años) (n = 102)a	Media (DS)b	24,6 (4,1)	-
	Rango	18-41	-
Estrato socioeconómico (n = 110)c	Uno	71	64,5
	Dos	32	29,2
	Tres	4	3,6
	Cuatro	2	1,8
	Cinco	1	0,9
Nivel educativo (n = 108)d	Primaria	12	11,1
	Secundaria	82	75,9
	Universitario	14	13,0

*a Nueve datos perdidos por no respuesta de las participantes; b Desviación estándar; c Un dato perdido por no respuesta de la participante; d Tres datos perdidos por no respuesta de las participantes.*

### Evaluación del estado serológico IgG e IgM frente a Toxoplasma gondii.

El análisis de respuesta humoral IgG específica para *T. gondii* reveló seropositividad en un 35,1% de la población (39 gestantes) y seronegatividad en el 64,9% (72 gestantes). Por su parte, la respuesta IgM resultó positiva en el 19,8% (22 gestantes) y negativa en el 80,2% (89, gestantes) de la población evaluada. A su vez, el 41% de las 22 participantes seropositivas para IgM, lo fueron concomitantemente para IgG (9 gestantes). (Tabla 2) En general, tomando en cuenta la detección de cualquiera de las dos inmunoglobulinas (o ambas simultáneamente), el 46,8% (52 casos) de la población resultó seropositiva al parásito. El análisis de la respuesta serológica teniendo en cuenta las dos inmunoglobulinas se muestra en la Tabla 2.

**Tabla 2:** Subgrupos poblacionales según respuesta serológica a *T.gondii* de mujeres gestantes.

Resultado para IgMa	Resultado para IgG	Estado serológico	Frecuencia absoluta (#)	Frecuencia relativa (%)
(-)	(-)	Seronegativa	59	53,2
(+)	(-)	Posible Primoinfecciónb	13	11,7
(+)	(+)	Posible Primoinfección/ Reinfecciónc		
(-)	(+)	Memoria Inmunológica	30	27,0
	Total		111	100

*aBasado en análisis semicuantitativo mediante inmunoensayo LIA (Line Immuno Assay); bInfección primaria; c Infección secundaria.*

Los resultados positivos para IgM fueron reportados de manera inmediata a la Coordinación de Control prenatal de la Institución de Salud vinculada el estudio, para efecto de la gestión de confirmación diagnóstica, así como de posterior manejo de las gestantes, en el marco del Programa de Control Prenatal, según protocolo establecido.

**Figura 1:**
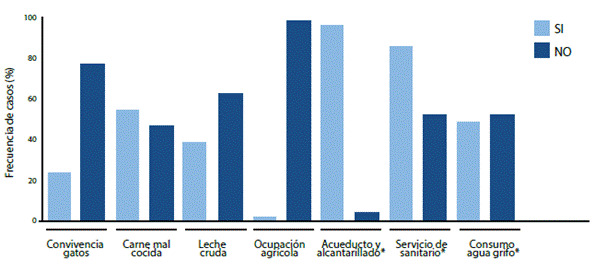
Descripción de factores de riesgo en población gestante de Institución de Salud, Cúcuta (Colombia), julio a octubre de 2018.

Por otra parte, el análisis descriptivo de factores de riesgo presentes en la población evaluada osciló entre 0 y 7, con predominio del consumo de carnes frescas (mal cocidas) en 60 casos (54,1% de las gestantes), seguido de consumo de leche cruda (cabra o vaca) en 44 casos (39,6% de las gestantes) y la convivencia con gatos en 26 (23,4%) de las gestantes (Figura 1 y Tabla 3); cabe mencionar que la mayoría de este último grupo (92%, 24 participantes) convivía sólo con un gato. En general se encontró que el 59,5% de las gestantes estaba expuesta a dos o más factores de riesgo.

Las barras representan la frecuencia relativa (n = 111) de exposición o no a diversos factores de riesgo en mujeres de primer trimestre de embarazo, entrevistadas mediante encuesta. *Aspectos evaluados sobre n = 110 (un caso sin respuesta a encuesta). Se empleó software SPSS v24.

**Tabla 3:** Asociación entre factores de riesgo y estado serológico IgG frente a *T.gondii* de mujeres gestantes. Seroprevalenciaa en Seroprevalenciaa en OR

Factor de riesgo	población expuesta n/N (%)	población no expuesta n/N (%)	(rango IC =95%)	Valor p
Convivencia con gatos	14/26 (53,8)	25/85 (29,4)	2,8 (1,1-6,9)	0,025
Consumo de carne mal cocida	25/60 (41,7)	14/51 (27,4)	1,8 (0,8-4,2)	0,120
Consumo de leche cruda de cabra/vaca	14/44 (31,8)	25/67 (37,3)	0,78 (0,3-1,7)	0,553
Consumo de agua del grifo	21/54 (38,9)	18/57 (31,6)	1,4 (0,6-3,0)	0,421
Servicio básico de acueducto	2/5 (40%)	37/106 (35,0)	0,8 (0,12-5,0)	0,816

ª*Basado en positividad para IgG específica anti-T.gondii mediante análisis semicuantitativo por inmunoensayo LIA (Line Immuno Assay).*

Se quiso caracterizar el agua de consumo dependiendo de la fuente de acceso, encontrándose que casi la mitad de la población, 54 gestantes (48,6% de la población evaluada), tomaban agua directamente de la llave (potable, procedente de planta de acueducto, pero sin algún otro tratamiento), y sólo menos de una tercera parte, 31 gestantes (27,9% de la población evaluada), lo hacía a partir de agua considerada como apta y lista para consumo humano, como la envasada (Figura suplementaria 1).

**Figura suplementaria 1.**
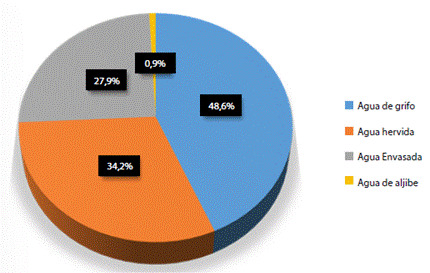
Descripción tipo de agua de consumo en población gestante de Institución de Salud, Cúcuta (Colombia), julio a octubre de 2018.

*El diagrama circular representa la frecuencia relativa de mujeres gestantes con acceso a distintas fuentes de agua para consumo permanente (n = 110). Se empleó software SPSS v24*

Finalmente, se evaluó la correlación entre la existencia de dos o más factores de riesgo con la positividad para alguna de las inmunoglobulinas, encontrando que, aunque la mayoría cumplía con este parámetro, no se pudo concluir que existiera asociación estadísticamente significativa (p= 0,558). Sin embargo, la presencia de dos o más factores de riesgo se observó en la totalidad de las gestantes IgM+IgG+ y en la mayoría de aquellas que evidenciaron memoria inmunológica, siendo sólo IgG+ (73,3%).

El análisis correlacional para los diversos factores de riesgo respecto al hallazgo de seropositividad IgG específica, evidenció asociación estadísticamente significativa con relación a la convivencia con gatos (p < 0,05) (Tabla 3).

## Discusión

La toxoplasmosis es una zoonosis de distribución mundial, cuyo agente etiológico es *Toxoplasma gondii*, un parásito intracelular ubicuo y que afecta a la tercera parte de la población humana mundial, además de otros mamíferos^17^^,^^30^. La transmisión ocurre a través de diversas fuentes, como lo son la ingesta de ooquistes fecales arrojados por los felinos, que contaminan el ambiente, la tierra, vegetales y el agua, quistes tisulares en las carnes de animales infectados, mucho menos probable taquizoitos en leche o cruda y la transmisión materno-fetal^3^^,^^31^^,^^32^, por lo que reviste especial relevancia la infección en población susceptible como las mujeres gestantes ocasionando ya sea aborto, parto pre-término o alteraciones neurológicas y oftalmológicas como retinopatía, de manifestación temprana o tardía en el recién nacido o posteriormente^33^^-^^35^, existiendo una mayor posibilidad de transmisión durante el último trimestre de embarazo, aunque una mayor afectación cuando la transmisión materno-fetal ocurre durante el primero^36^^,^^37^.

En tal sentido, el tamizaje serológico para anticuerpos anti-*T.gondii* durante el control prenatal ha sido adoptado como medida preventiva en diversos países, incluida Colombia, en el marco de la Política de Atención Integral en Salud, obligatoria en el ámbito nacional y enfocada a la evaluación inicial durante el primer trimestre, pero con periodicidad mensual ante hallazgo de seronegatividad ^22^^,^^23^^,^^38^^,^^39^.

Su diagnóstico requiere de una combinación de análisis epidemiológicos y clínicos, dado a que esta infección es una enfermedad desatendida y no existe un procedimiento estándar para la realización de los servicios de atención médica^40^, por lo que a nivel prenatal se basa en la detección de inmunoglobulinas en la gestante, inicialmente cuantificando la IgM en relación a infección reciente, pese a que su elevación puede mantenerse varios meses post-infección, como se reconoce desde hace varias décadas^27^^,^^28^. En tal sentido, en el presente estudio se realizó detección de IgM específica empleando un test basado en antígeno de fase temprana ROP1c de *T. gondii*, reflejando posible infección alrededor de las últimas ocho semanas.

En la presente investigación encontramos que un poco más de la mitad de las gestantes (53,2%) no evidenció respuesta humoral alguna contra el parásito (seronegatividad), lo que podría implicar riesgo de contraer la infección durante la gestación si se tiene en cuenta que se halló un 60% de la población expuesta a mínimo dos factores de riesgo relacionados. Sumado a lo anterior, el 11,7% de los casos evaluados (13 gestantes) resultaron compatibles con infección muy reciente (positividad sólo para IgM, Tabla 2), lo cual se traduce en posibilidad ya sea de infección como de transmisión al feto durante la gestación, representando sin duda un reto al fortalecimiento del sistema de vigilancia para toxoplasmosis gestacional a través del control prenatal^23^, aspecto que podría evaluarse a partir de un nuevo estudio de seguimiento al desenlace de los casos contemplados en el marco del presente trabajo, dado que dicho reconocimiento se encuentra fuera del alcance inicial propuesto y aceptado para el mismo, enfocado en la evaluación de población gestante durante su primer control prenatal (primer trimestre de embarazo). Este tipo de esfuerzos podrían precisamente cimentar una vigilancia integral del evento toxoplasmosis gestacional con interacción entre instituciones de salud y comunidad científica.

En consistencia con lo anterior, la frecuencia de casos con sospecha de infección reciente medida a través de la positividad para IgM específica en el presente estudio (en total 19,8% de los casos) fue superior a la reportada para población gestante en nuestra región representando casi 7 veces la frecuencia referida previamente (3%)^41^, así como a nivel nacional (1,6- 6,1%)^42^^,^^43^ e internacional, como 2,3-4% (toda edad gestacional)^44^^,^^45^; incluso en población en general aparentemente sana se ha reportado recientemente desde 0,6-11,3% de seropositividad asociada a infección activa en donantes de sangre^46^^,^^47^, datos todos en contraste con la elevada seroprevalencia IgM hallada en nuestra población evaluada, siendo superados sólo por un estudio en mujeres gestantes, a partir de un grupo preseleccionado con sospecha de toxoplasmosis aguda en Italia (n = 302), donde el 57% resultó seropositivo para IgM anti-*T.gondii* mediante inmunofluorescencia indirecta^39^.

Por su parte, determinamos respuesta IgG específica en el 35,1% de la población evaluada, en consistencia con un estudio previo en mujeres gestantes realizado por nuestro grupo de investigación en a nivel local (31,1%)^41^, así como la registrada en otros países suramericanos (39%)^48^^,^^49^ e incluso en otros continentes como el Asiático, a partir de población general (34,5%)^46^ y de mujeres gestantes (32,4%)^44^, aunque superior a la referida en ésta misma población a nivel nacional (28,2% en Bogotá)^43^ e internacional en otras regiones tropicales como India (26%)^45^, Etiopía (24%)^50^ y no tropicales como Estados Unidos (6-9% de mujeres en edad fértil)^51^.

Sumado a los anteriores hallazgos sobre seroprevalencia, el presente estudio reveló exposición a dos o más factores de riesgo relacionados en el 60% de las gestantes evaluadas, cifra elevada y contrastante con la referida en otros países donde no supera el 50%^51^, resultando preocupantes aspectos como el consumo de carme mal cocida y de agua de la llave sin ningún otro procesamiento en el 54,1 y 48,6% de la población analizada, respectivamente, pese a no hallarse directamente asociación estadística con seroprevalencia IgG al parásito, hecho que refleja un componente cultural.

En tal sentido, sorprende encontrar que predominó esta opción (casi la mitad de las participantes consumen agua directamente del grifo) frente a otras como agua envasada o hervida; por lo que deseamos resaltar que aunque no se encontró asociación significativa con la seroprevalencia, sí encontramos una mayor frecuencia de seropositividad frente al parásito en mujeres expuestas (Tabla 3), considerando relevante describir este aspecto en busca de brindar opciones de fortalecimiento del control prenatal a partir estrategias como la capacitación hacia el consumo de agua segura o apta para tal fin durante la gestación, por ejemplo, toda vez que se contribuya a resguardar la salud integral materno-fetal (Figura suplementaria 1).

En contraste, este último factor se ha encontrado asociado previamente en otras regiones del país, según lo referido para un grupo de gestantes de Armenia, Colombia, donde el consumo de bebidas a base de agua sin hervir representó un OR de 4,5 (p = 0,01), mientras que el consumo de agua tratada y envasada constituyó un factor protector^52^; también en otros países en vía de desarrollo (Etiopía, África) éste tipo de exposición ha resultado relevante, donde el consumo de agua no tratada se asoció a seropositividad IgG específica (p = 0,08)^50^ e incluso con el riesgo referido en Europa en la región Champagne-Ardenne al nororiente de Francia, donde se detectó ADN del parásito en 10 de 125 muestras de agua de acceso público en un estudio longitudinal de 2001 a 2003^53^.

Se ha considerado incluso un riesgo indirecto el empleo de agua del grifo para el lavado de frutas o vegetales frescos^5^^,^^54^, lo que ha llevado a sugerir la remoción de la cáscara de éstos alimentos como medida protectora^55^, por lo que dicho aspecto, sin embargo, no debe pasar desapercibido y continuar en seguimiento mediante estudios futuros, que permitan dilucidar el posible rol del agua de acceso humano respecto al evento toxoplasmosis en nuestra región, pues dicha agua procede de sistemas de acueducto local y del área metropolitana.

Por su parte, la exposición a otros factores de riesgo como consumo de carne mal cocida fue relevante en nuestro estudio puesto que abarcó más de la mitad de la población evaluada (54,1% casos) aunque no reveló asociación con seropositividad, en contraste con lo referido por López y colaboradores en Armenia, Colombia, donde precisamente éste tipo de exposición ha mostrado ser una gran problemática^52^ o bien en Estados Unidos u otros países^3^^,^^9^.

Nuestros resultados señalan una asociación significativa entre la convivencia con gatos (OR=2,8, p < 0,05, IC= 95%) y el riesgo de infección por *T. gondii* en nuestra población, consistente con la relación atribuida al contacto con estos felinos^45^^,^^52^, pese a que sólo una cuarta parte de la población gestante analizada estaba expuesta al contacto permanente con gatos y a que una amplia mayoría de estos 26 casos (92%) convivía con sólo uno de estos animales.

De modo relevante, el 40% de las gestantes evaluadas manifestó consumir leche cruda, factor que aunque no se encontró asociado a positividad para IgG específica en la presente investigación, vale la pena considerar en posteriores estudios, teniendo en cuenta el agravante que representa la presencia demostrada del parásito en leche cruda de cabra en Brasil (detección de ADN del parásito) y Egipto, aunque a bajas proporciones (inferiores al 4%)^56^^,^^57^, antes de lo cual había sido referida la asociación del consumo de leche no pasteurizada de cabra a riesgo de infección reciente por *T.gondii* en población humana adulta, incluyendo mujeres gestantes, en Estados Unidos (OR: 5,09)^58^.

Vale la pena resaltar que el riesgo lo representa además del consumo de la leche, el de sus derivados, puesto que muy recientemente ha sido referida la detección del parásito a partir de queso fresco artesanal, obtenido a partir de leche de vaca, durante un brote de toxoplasmosis ocurrido en el año 2016 en Goiás, centro occidente de Brasil, donde el 79% de los casos estuvieron expuestos a dicho consumo^5^. Puesto que se trata del primer posible brote asociado a este factor de riesgo, como especifican los autores, aunque no se logró establecer plenamente relación de causalidad, si refieren contacto de la mayoría de los casos de toxoplasmosis aguda identificados (11/14 personas) con la fuente de riesgo (consumo de queso artesanal) resaltando dos factores relevantes, la leche cruda como insumo y la posible contaminación con ooquistes del parásito durante el proceso de manufactura.

A diferencia de continentes como Europa, Asia y África, un metaanálisis reciente reveló que en países de Norte y Latinoamérica si existe correlación entre el consumo de leche cruda de cabra y riesgo de toxoplasmosis en humanos^59^, con el agravante de la demostrada capacidad de supervivencia de los taquizoitos de *T.gondii* en leche cruda^60^, incluso en presencia de jugo gástrico^61^, por lo que resulta necesario establecer la relación de ésta variable con la respuesta humoral asociada a infección aguda, confirmada idealmente mediante prueba de avidez IgG.

## Conclusiones

Los hallazgos de la presente investigación revelan tanto una alta posibilidad de contacto con *T.gondii* en la población gestante evaluada, ratificando la asociación de la convivencia con gatos (incluso con uno solo) con este evento, como también un panorama de exposición a diversos factores de riesgo relacionados a toxoplasmosis, centrados en el consumo de fuentes alimenticias contaminadas, especialmente agua directamente del grifo (definida como potable pero aun así, considerada no apta para el consumo humano directo) y carne mal cocida, los cuales, aunque no se correlacionaron estadísticamente a respuesta IgG específica en la muestra poblacional evaluada, si reflejaron una mayor seropositividad en población expuesta (respecto a no expuesta; Tabla 3), permitiéndonos por una parte, reiterar la importancia atribuida a los alimentos como vía de transmisión de *Toxoplasma gondii*^62^ y por ende del riesgo de enfermedad asociada, con consecuencias como discapacidad, pérdida de la calidad de vida o muerte^48^^,^^63^^,^^64^, y por otra, vislumbrar la necesidad de futuras investigaciones que contemplen un número más amplio de participantes, en torno a evaluar la asociación de estos factores de riesgo tanto frente a la respuesta asociada a memoria inmunológica, como frente a casos de infección reciente ya confirmados serológicamente (considerando que el número limitado de gestantes evaluadas en el presente pudo afectar la significancia estadística frente a factores relevantes por su comportamiento entre los grupos expuesto y no expuesto, lo cual representa una limitante del presente estudio).

Por lo tanto, se requiere de un fortalecimiento de las estrategias de abordaje integral de la mujer embarazada durante el control prenatal, incluyendo el reconocimiento y prevención de factores de riesgo en suma al seguimiento y diagnóstico temprano de toxoplasmosis gestacional, toda vez que la detección de IgG en la madre (en relación a memoria inmunológica) constituye un factor protector para el feto al protegerle de infección congénita y sus consecuencias neurológicas^65^, confiriendo a la vigilancia serológica de rutina un rol importante en el reconocimiento del estado de exposición a este evento infeccioso en las madres^51^, la cual se recomienda sea lo más periódica posible teniendo en cuenta que el esquema trimestral propuesto y vigente en diversos países es considerado generar periodos ciegos en el seguimiento de la gestante y por ende en el riesgo de transmisión al feto, especialmente hacia el final de la gestación^66^.

Dicho fortalecimiento debe tomar en cuenta la importancia de la educación de las mujeres gestantes acerca de estrategias de prevención, así como realizarse seguimiento a su implementación por parte del equipo de salud, toda vez que algunos antecedentes de consulta en Latinoamérica sobre conocimiento de la infección (fuente, vías de transmisión, prevención y síntomas) en mujeres gestantes y población femenina en edad fértil, han arrojado resultados notablemente no satisfactorios^67^, lo que resulta preocupante y sugiere, en tal sentido, la adopción de modelos de países europeos como Francia, Austria e Italia, donde el bioanálisis realizado inicialmente durante el primer trimestre, se continúa necesariamente tras cada mes de gestación, a través de un abordaje integral que incluye capacitación o consejería de la mujer gestante en torno a la prevención del riesgo de contaminación, entre las estrategias que buscan controlar el evento^68^.

Tomando todo, como fortaleza la presente investigación visibiliza la alta exposición a factores de riesgo de infección con *T.gondii* en mujeres gestantes de primer trimestre, conjuntamente con reactividad IgM en una quinta parte de una muestra poblacional pequeña, situación considerada grave ya que que podría constituir un reflejo del panorama actual de este evento infeccioso en nuestra región fronteriza, invitando tanto a la investigación futura y permanente como a la necesidad de considerar estrategias de intervención en la población gestante a través de capacitación continua frente a la infección y el riesgo de enfermedad fetal por su transmisión, desde un contexto preventivo.

El presente estudio estuvo sujeto a las consideraciones expuestas en la resolución Nº 8430 de 1993 del Ministerio de Salud de Colombia, teniendo en cuenta lo contemplando en el Título II, Capitulo 1 “de los aspectos éticos de la investigación en seres humanos", Artículo 11 donde se establece un riesgo mínimo para los participantes basado en extracción venosa a bajos volúmenes^69^, procedimiento realizado en única ocasión e implementado en las condiciones de bioseguridad requeridas y por medio de personal experto.
